# Physical activity and nutrition behavioural outcomes of a home-based intervention program for seniors: a randomized controlled trial

**DOI:** 10.1186/1479-5868-10-14

**Published:** 2013-01-31

**Authors:** Linda Burke, Andy H Lee, Jonine Jancey, Liming Xiang, Deborah A Kerr, Peter A Howat, Andrew P Hills, Annie S Anderson

**Affiliations:** 1 School of Public Health, Curtin University, Perth, WA, Australia; 2 School of Physical and Mathematical Sciences, Nanyang Technological University, Nanyang, Singapore; 3 Centre for Behavioural Research in Cancer Control, Curtin University, Perth, WA, Australia; 4 Mater Mother’s Hospital/Mater Medical Research Institute (MMRI), Griffith University/Griffith Health Institute (GHI), Griffith, QLD, Australia; 5 Centre for Public Health Nutrition Research, Division of Clinical Population Sciences and Education, University of Dundee, Scotland, UK

**Keywords:** Fat avoidance, Fibre intake, Fruit intake, Goal setting, Sitting, Strength exercise, Vegetable intake, Walking

## Abstract

**Background:**

This intervention aimed to ascertain whether a low-cost, accessible, physical activity and nutrition program could improve physical activity and nutrition behaviours of insufficiently active 60–70 year olds residing in Perth, Australia.

**Methods:**

A 6-month home-based randomised controlled trial was conducted on 478 older adults (intervention, n = 248; control, n = 230) of low to medium socioeconomic status. Both intervention and control groups completed postal questionnaires at baseline and post-program, but only the intervention participants received project materials. A modified fat and fibre questionnaire measured nutritional behaviours, whereas physical activity was measured using the International Physical Activity Questionnaire. Generalised estimating equation models were used to assess the repeated outcomes over both time points.

**Results:**

The final sample consisted of 176 intervention participants and 199 controls (response rate 78.5%) with complete data. After controlling for demographic and other confounding factors, the intervention group demonstrated increased participation in strength exercise (p < 0.001), walking (p = 0.029) and vigorous activity (p = 0.015), together with significant reduction in mean sitting time (p < 0.001) relative to controls. Improvements in nutritional behaviours for the intervention group were also evident in terms of fat avoidance (p < 0.001), fat intake (p = 0.021) and prevalence of frequent fruit intake (p = 0.008).

**Conclusions:**

A minimal contact, low-cost and home-based physical activity program can positively influence seniors’ physical activity and nutrition behaviours.

**Trial registration:**

anzctr.org.au Identifier: ACTRN12609000735257

## Background

Physical activity is known to decline with age [1]. In Australia, 51% of the older population aged 60 to 75 years are insufficiently active, with the highest prevalence of inactive behaviour being reported in adults over 75 years of age [2]. Similarly, rates of physical activity among American adults aged 65 years and older are low, with only 20% of women and 25% of men meeting the national recommended physical activity guidelines [3], while 26% of those in the 65–74 age group are inactive [4]. Research has demonstrated that sedentary behaviours may be linked to obesity, cardiovascular diseases and type 2 diabetes [5-11]. Moreover, as people age, their nutritional requirements change and energy requirements decrease. Older adults should consume nutritious foods that are high in fibre and low in saturated fats to help maintain a healthy weight [12]. However, worldwide trends are shifting towards an increased consumption of energy-dense foods rich in saturated fats and sugars [13], leading to energy imbalance and rise in diet related diseases [14].

In the literature, intervention programs designed to improve physical activity levels or dietary habits have used a variety of strategies including workbooks, calendars, telephone counselling, goal setting and pedometers [15-19]. Although interventions combining physical activity and nutrition appear to result in better outcomes than those focusing on either aspect alone [17,19], there is limited evidence on home-based interventions in terms of improving both physical activity and nutritional behaviours among people aged 60–70 years [20-22]. Moreover, research involving seniors has generally been undertaken with a small sample size [18,19,23], or targeting those with a specific chronic disease [15,19,23]. Another limitation is that the participants recruited were generally self-referrals and volunteers [17,18,24] as opposed to being randomly selected samples. Therefore, there is an urgent need to develop well-designed interventions that overcome such shortcomings [25].

The Physical Activity and Nutrition for Seniors (PANS) program attempted to improve both physical activity and nutritional behaviours. It was a low-cost and accessible home-based intervention targeting insufficiently active low to middle income older adults aged 60–70 years who could semi-tailor the program to suit their own pace and needs [26]. We targeted these “baby boomers” (60–70 year olds) because they contribute to the fast growing segment of the population who are retired or near retirement. The aim of the present study was to determine whether the PANS intervention was effective with respect to the main outcome measures of self-reported physical activity and nutritional behaviours. The findings have important implications for the control and prevention of overweight and obesity in the older population.

## Methods

### Study design

PANS was a 6-month two-arm randomised controlled trial collecting data at two time points (baseline; post intervention). The project protocol was approved by the Human Research Ethics Committee of Curtin University (approval number HR 186/2008) and written consent was obtained from all participants.

### Procedure

**Figure 1:**
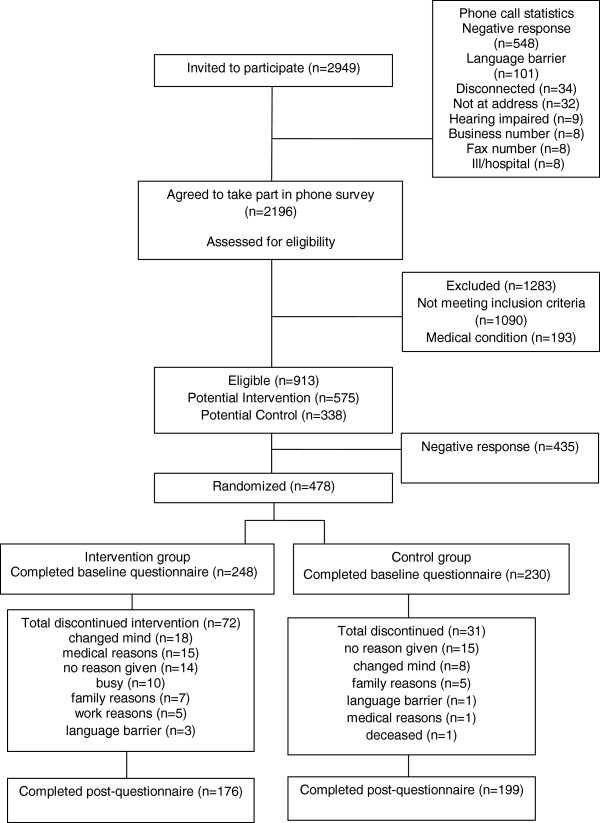
Consort flow chart of intervention participants and controls.

A random sample of 478 participants was recruited from 60 suburbs/neighbourhoods (30 intervention; 30 controls) within the metropolitan area of Perth, the capital of Western Australia. Participants were randomly selected from the Australian Federal Electoral Roll in 2010, which provided a representative sampling frame. Suburbs were required to comprise at least 14% of adults aged 60 years and older; contain at least 120 potential participants; and be classified as low or medium socio-economic status [27]. Participant selection criteria called for “insufficiently active” recruits who participated in less than 30 minutes of moderate-intensity physical activity on at least 5 days per week [28]; aged 60 to 70 years; considered “healthy” to the extent that participation in a low-stress physical activity program would not place them at risk; and not on any special diet [26]. Participant flow and corresponding sample sizes are presented in Figure 1. Of the 248 program participants and 230 controls that completed the baseline questionnaire, 176 and 199 seniors respectively with complete data were available for analysis, giving a final response rate of 78.5%.

### Intervention

The intervention was developed using Social Cognitive Theory [29,30] and the Precede-Proceed Model [31] incorporating voluntary cooperation and self-efficacy [29,32], and based on a pilot project [33] that produced encouraging results with respect to adherence and behaviour change [33,34]. Further revisions were made after extensive formative research [26]. Such formative data from representatives of the target group confirmed the preference for a flexible, home-based program, whereby participants would be able to set their own goals and semi-tailor the intervention to better suit their own needs [26].

The main resource of the home-based program was a booklet specially designed for seniors that provided physical activity and nutrition recommendations and encouraged goal setting. The booklet was supported by an exercise chart, calendar, bi-monthly newsletters, resistance band and pedometer, along with telephone and email contact by program guides. Frequency of telephone contact varied, as some participants requested only phone contact or information via email. Participants generally received between six to 10 phone calls and/or two to five emails over the 6-month period. The protocol of the intervention has been described in detail elsewhere [26]. The control group received baseline and post-intervention questionnaires at the same time as the PANS participants, and both groups were given a small token of appreciation upon completion and return of the postal questionnaires.

The intervention was funded for A$400,000 over a three-year period. The estimated costs to replicate the intervention include salary for a part-time coordinator (A$180,000) and a research assistant (A$150,000), intervention materials (A$23,500) and incentives (A$7,000), postage (A$1,500), telephone calls (A$3,500), program guide reimbursement (A$6,500), guide manuals (A$500), administration costs (A$5,000) and printing of questionnaires (A$2,500).

### Instrument

The self-completion questionnaire consisted of previously validated instruments on physical activity and sitting behaviour [35] and nutrition behaviours [36], along with demographic and personal characteristics including gender, age, education level, marital status, tobacco smoking and alcohol consumption. The instrument was reviewed by experts in the field, underwent test re-test, and found to possess moderate to high intra-class correlation (0.62-0.95). The International Physical Activity Questionnaire short-form [35] was used to measure self-reported walking, moderate-intensity physical activity, vigorous-intensity physical activity and sitting time for older adults [37,38]. It specifically asked whether a person participated (yes or no) in various types of physical activity and their duration (minimum of 10 minutes). A strength exercise question “During your usual week, on how many days did you do strength activities? How much time did you usually spend doing strength activities on each of these days?” was also appended [39].

Dietary intake behaviours were assessed via a modified version of the Fat and Fibre Barometer [36] to gather specific information on fat intake (e.g. butter, cheese, milk) and fibre-related intake (e.g. cereals, fruit and vegetables). Extra questions were added to assess frequency of fruit and vegetable intake, which enabled quantification of the number of days participants consumed at least two servings of fruit or vegetables per week. The content of the intervention emphasised increasing consumption of fruits, vegetables and fibre but reducing the intake of saturated fat.

### Statistical analysis

Descriptive statistics were first applied to summarize the baseline demographic profile and lifestyle characteristics of the sample. Comparisons between intervention and control groups were made across the two time points using independent samples and paired t-tests for continuous outcomes, and chi-square test for categorical outcome variables.

The main outcomes of interest were strength exercise, walking, moderate- and vigorous-intensity physical activity levels, sitting time, fibre intake, fat intake, fat avoidance, frequency of fruit intake and frequency of vegetables intake. In the presence of many zeros (lack of participation by seniors, i.e. < 10 minutes duration), all physical activity variables were recoded into binary form indicating participation status (yes; no), while sitting time remained as a continuous variable (recorded in minutes per week). For food eating habits, the fibre intake (range 0–28), fat intake (range 0–21) and fat avoidance (range 6–30) composite scores were computed based on the corresponding consumption behavioural questions from the Fat and Fibre Barometer, whereas consumption of at least two servings of fruit per week was considered as either infrequent (0 to 2 days) or frequent (3 to 7 days), and analogously for vegetables consumption.

To accommodate the inherent correlation of observations taken from the same individual, generalized estimating equation (GEE) models with exchangeable correlation structure were fitted to assess the repeated measures over time, while accounting for the effects of potential confounding factors. All binary outcomes were modelled using logistic GEE. Normal GEE with identity link was applied to fibre intake and fat intake scores, whereas gamma GEE with log link was considered appropriate for modelling the highly skewed sitting time variable and fat avoidance score. All statistical analyses were undertaken in the SPSS package, version 18.

## Results

Characteristics of the sample are summarized in Table 1 which shows the intervention and control groups were similar in terms of demographics and lifestyle at baseline. Overall, the mean age was 66 years, about half were male, the majority of them had a partner and experienced common health conditions. Less than half the seniors completed secondary school but over 40% were still in the workforce. No differences in reported alcohol drinking and smoking status were found between the intervention and control participants. No adverse events were reported in relation to the intervention.

**Table 1:** Baseline characteristics of intervention participants and controls

**Variable**	**Intervention group (n = 176)**	**Control group (n = 199)**	***p *value **^**1**^
Age: mean (SD) years	65.80 (2.95)	65.75 (3.19)	0.884
Gender: male	93 (52.8%)	101 (50.8%)	0.686
Relationship status: with partner	128 (72.7%)	159 (79.9%)	0.102
Work status: working	77 (43.8%)	80 (40.2%)	0.487
Co-morbidity ^2^: yes	129 (73.3%)	139 (69.8%)	0.461
Education level: primary school	8 (4.5%)	16 (8.0%)	0.483
secondary school	83 (47.2%)	91 (45.7%)	
trade certificate/diploma	48 (27.3%)	57 (28.6%)	
university	37 (21.0%)	35 (17.6%)	
Financial struggle: never	24 (13.6%)	25 (12.6%)	0.951
sometimes	115 (65.3%)	131 (65.8%)	
always	37 (21.0%)	43 (21.6%)	
Alcohol drinking: yes	116 (65.9%)	137 (68.8%)	0.545
Smoking status: never	97 (55.1%)	94 (47.2%)	0.283
former	69 (39.2%)	94 (47.2%)	
current	10 (5.7%)	11 (5.5%)	

^1^ chi-square or t test between intervention and control groups.

^2^ presence of at least one of nine common health conditions.

Process evaluation based on a brief questionnaire indicated good adherence to the program. Participants reported that the booklet encouraged them to think about physical activity (78%) and nutrition (70%), with the majority using the exercise chart (74%) to practise the recommended exercises (62%). Moreover, the calendar reminded them to consider physical activity (66%) and nutrition (55%). About 90% of the intervention participants reported using the pedometer while 63% utilised the resistance band to perform strength exercises.

Table 2 compares the physical activity outcomes between the intervention and control groups across the two time points. Both groups were similar in terms of physical activity participation at baseline, except in sitting time. However, significant improvements in these outcomes from baseline to post-program were evident among the intervention participants but not the controls. In particular, the intervention group exhibited significantly higher prevalence of participation in strength exercise and walking than the control group at six months.

**Table 2:** Comparison of physical activity outcomes between intervention participants and controls

**Outcome**	**Intervention group (n = 176)**		**Control group (n = 199)**		**chi-square or t test**
	**Baseline**	**Post**	**Baseline**	**Post**	
Strength exercise ^1^	34 (19.3%)	70 (39.8%)	55 (27.6%)	55 (27.6%)	*p*_2_ = 0.060 *p*_3_ = 0.013
	*p*_1_ < 0.001		*p*_1_ = 1		
Walking ^1^	152 (86.4%)	166 (94.3%)	171 (85.9%)	173 (86.9%)	*p*_2_ = 0.903 *p*_3_ = 0.015
	*p*_1_ = 0.012		*p*_1_ = 0.770		
Moderate activity ^1^	124 (70.5%)	145 (82.4%)	143 (71.9%)	154 (77.4%)	*p*_2_ = 0.764 *p*_3_ = 0.229
	*p*_1_ = 0.008		*p*_1_ = 0.205		
Vigorous activity ^1^	33 (18.8%)	49 (27.8%)	55 (27.6%)	51 (25.6%)	*p*_2_ = 0.050 *p*_3_ = 0.629
	*p*_1_ = 0.044		*p*_1_ = 0.650		
Sitting time: mean (SD) min per week	2063 (1050)	1708 (952)	1691 (925)	1734 (986)	*p*_2_< 0.001 *p*_3_ = 0.794
	*p*_1_ < 0.001		*p*_1_ = 0.441		

^1^ participation of at least 10 minutes.

*p*_1_ : baseline versus post *p* value.

*p*_2_ : baseline intervention versus baseline control *p* value.

*p*_3_ : post intervention versus post control *p* value.

Comparison of nutritional behaviours are summarised in Table 3. All nutritional outcomes were similar between the two groups at baseline. At six months, the intervention participants demonstrated significant increases in fibre intake and fat avoidance, with significantly higher mean scores than the controls. Improvements in prevalence of frequent fruit intake and prevalence of frequent vegetable intake were also observed in the intervention group, though the latter increase appeared marginal. As expected, there was little change in dietary habits among the controls over the six-month period.

**Table 3:** Comparison of nutritional outcomes between intervention participants and controls

**Outcome**	**Intervention group (n = 176)**		**Control group (n = 199)**		**chi-square or t test**
	**Baseline**	**Post**	**Baseline**	**Post**	
Frequent fruit intake ^1^	153 (86.9%)	164 (93.2%)	167 (83.9%)	163 (81.9%)	*p*_2_ = 0.250 *p*_3_ = 0.001
	*p*_1_ = 0.037		*p*_1_ = 0.345		
Frequent vegetable intake ^1^	155 (88.1%)	165 (93.8%)	170 (85.4%)	177 (88.9%)	*p*_2_ = 0.275 *p*_3_ = 0.072
	*p*_1_ = 0.047		*p*_1_ = 0.184		
Fibre intake score: range 0–28, mean (SD)	16.77 (5.60)	18.07 (5.30)	16.14 (6.05)	16.74 (6.05)	*p*_2_ = 0.300 *p*_3_ = 0.025
	*p*_1_< 0.001		*p*_1_ = 0.035		
Fat avoidance score: range 6–30, mean (SD)	21.53 (4.83)	22.81 (4.34)	21.36 (4.78)	21.49 (4.77)	*p*_2_ = 0.757 *p*_3_ = 0.009
	*p*_1_< 0.001		*p*_1_ = 0.953		
Fat intake score: range 0–21, mean (SD)	1.84 (1.99)	1.63 (1.60)	1.47 (1.56)	1.60 (1.86)	*p*_2_ = 0.280 *p*_3_ = 0.350
	*p*_1_ = 0.049		*p*_1_ = 0.230		

^1^ consumption of at least two servings on 3 to 7 days per week.

*p*_1_ : baseline versus post *p* value.

*p*_2_ : baseline intervention versus baseline control *p* value.

*p*_3_ : post intervention versus post control *p* value.

Results of the GEE analyses are given in Table 4. After controlling for demographic and other confounding factors, the regression results confirmed significant increases in engagement in strength exercise (p < 0.001), walking (p = 0.029) and vigorous-intensity physical activity (p = 0.015) but not moderate-intensity physical activity (p = 0.144) for the intervention participants relative to the controls. The PANS intervention was also successful in significantly reducing the sitting time of participants through the group×time interaction term (p < 0.001). Moreover, positive behavioural changes towards reducing dietary fats were evident in the intervention group in terms of fat avoidance (p < 0.001) and fat intake (p = 0.021) when compared with the controls. The likelihood of frequent fruit intake significantly increased among the PANS participants post-intervention (p = 0.008), but fibre intake and prevalence of frequent vegetable intake did not change significantly after the six-month period. The estimated correlations between the repeated observations were substantial which justified the fitting of GEE models.

**Table 4:** Regression analysis of outcomes before and after intervention (n = 375)

**Outcome**	**Coefficient**^**1**^	**95% Confidence interval**	***p*value**	**Correlation**^**2**^
Strength exercise ^3^	1.075	(0.559, 1.591)	< 0.001	0.417
Walking ^3^	0.909	(0.094, 1.724)	0.029	0.314
Moderate activity ^3^	0.416	(−0.142, 0.974)	0.144	0.387
Vigorous activity ^3^	0.664	(0.128, 1.199)	0.015	0.405
Sitting time ^4^	−0.215	(−0.312, -0.117)	< 0.001	0.583
Frequent fruit intake ^3^	0.921	(0.236, 1.607)	0.008	0.400
Frequent vegetable intake ^3^	0.424	(−0.403, 1.251)	0.314	0.275
Fibre intake ^5^	0.716	(−0.115, 1.546)	0.091	0.742
Fat avoidance ^4^	0.057	(0.028, 0.085)	< 0.001	0.843
Fat intake ^5^	−0.345	(−0.639, -0.051)	0.021	0.637

^1^ effect of group by time interaction, adjusted for age, gender, relationship status, work status, co-morbidity, education level, financial struggle, alcohol drinking, smoking status, group (intervention/control) and time (baseline/post).

^2^ exchangeable correlation estimate.

^3^ logistic generalized estimating equation model.

^4^ gamma generalized estimating equation model with log link.

^5^ normal generalized estimating equation model with identity link.

## Discussion

Appropriate home-based interventions can improve physical activity and nutrition behaviours in insufficiently active 60–70 year olds [33,34,40], and are especially useful when they allow for flexibility, with self-tailoring to suit individual pace and needs [15,33,38]. The PANS intervention was developed based on a large pilot study [33,34] and offered a practical community-based program for older people. The relatively low cost trial was designed to evaluate the effect of combining physical activity and nutrition on behavioural changes of seniors with low to middle socioeconomic status. The moderate sample sizes provided sufficient statistical power for evaluation of the repeated measures [26]. The overall response rate of 78.5% was comparable with other randomized controlled trials on seniors [24,41]. The main reasons of attrition such as work and family commitments, illness and injuries, were consistent with other studies in the literature [15,24]. The International Physical Activity Questionnaire short-form appears to be useful to assess physical activity behavioural change for older adults. However, objective assessment of physical activity should be considered in future research.

The results from this 6-month home-based intervention for seniors indicated improvements in physical activity and nutritional behaviours among program participants in comparison to the controls. The intervention was shown to be effective and consistent with previous studies in terms of levels of change in physical activity and nutrition behaviours [15], specifically, increases in walking [34,38], participation in strength exercises [15], increases in vigorous-intensity physical activity [42], improvements in fruit intake [15,43] and a reduced consumption of fat [15]. However, fibre intake behaviour and the frequency of vegetable intake showed no significant change. The seniors may already maintain a varied and healthy diet with a low consumption of take-away foods at baseline. This could have imposed limitations on further dietary gains, producing a so called “ceiling effect” [34,44].

The health benefits of physical activity and its role in preventing many chronic diseases are well established [6,10,45]. On the other hand, recent research has suggested that sitting for long periods of time can have a detrimental effect on the body’s physiology, with excessive sitting being recognised as a serious health hazard [5]. The PANS intervention was effective in reducing the sitting time of seniors. There is clearly a need for incorporating sitting time within physical activity guidelines [3,46,47], and positive change in sedentary behaviour should be a key component of future intervention programs.

### Limitations

In this study, the data collected from the postal questionnaires were based on self-report, although similar inaccuracies would be expected between the intervention and control groups. Large scale community trials have used self-reported data as valid proxies to reduce cost and attrition rates, and such data have been considered sufficiently reliable for monitoring changes over time [15,48-51] which formed the basis of our evaluation. Self-selection bias was minimized through randomisation, but participation in the home-based intervention was entirely voluntary. Therefore, reporting bias might still be a problem. Furthermore, residual confounding could not be ruled out even though demographic and other factors were controlled for in the GEE regression analyses.

## Conclusions

The PANS participants improved their physical activity and dietary habits in comparison to the controls, confirming that a low-cost, home-based physical activity and nutrition program tailored for insufficiently active, low to middle income seniors can produce effective behavioural changes. A follow-up study is recommended to confirm the adherence of the positive behavioural changes beyond six months. It would also be useful to replicate the program both in the community and in other settings where seniors reside such as retirement villages.

## Abbreviations

GEE: generalized estimating equation; PANS: physical activity and nutrition for seniors.

## Competing interests

The authors declare that they have no competing interests.

## Authors’ contributions

LB conducted the trial and drafted the manuscript. PAH coordinated the study. PAH, AHL, JJ, DAK, APH and ASA contributed to conception and study design and revised the manuscript. LX and AHL performed statistical analysis and interpreted the data. All authors read and approved the final manuscript.
